# Effects of Peppermint Extract and Chitosan-Based Edible Coating on Storage Quality of Common Carp (*Cyprinus carpio*) Fillets

**DOI:** 10.3390/polym13193243

**Published:** 2021-09-24

**Authors:** Ana Gabriela Morachis-Valdez, Ángel Santillán-Álvarez, Leobardo Manuel Gómez-Oliván, Imelda García-Argueta, Hariz Islas-Flores, Octavio Dublán-García

**Affiliations:** 1 Laboratorio de Alimentos y Toxicología Ambiental, Facultad de Química, Universidad Autónoma del Estado de México, Paseo Colón intersección Paseo Tollocan s/n. Col. Residencial Colón, Toluca 50120, Mexico; agmorachis@gmail.com (A.G.M.-V.); lgolivan74@gmail.com (L.M.G.-O.); hislasf@uaemex.mx (H.I.-F.); 2 División de Gastronomía, Tecnológico de Estudios Superiores de Valle de Bravo, Tecnológico Nacional de México, Km. 30, Carretera Federal Monumento Valle de Bravo, San Antonio de la Laguna, Valle de Bravo 51200, Mexico; angel.sa@vbravo.tecnm.mx; 3 Facultad de Medicina, Universidad Autónoma del Estado de México, Av. Paseo Tollocan 134 502, Residencial Colón y Col Ciprés, Toluca 50120, Mexico; igarciaa@uaemex.mx

**Keywords:** chitosan, peppermint extract, *Cyprinus carpio*, physicochemical properties, microbiological quality, carbonyl proteins groups

## Abstract

Edible coatings have recently been developed and applied to different food matrices, due to their numerous benefits, such as increasing the shelf life of foods, improving their appearance, being vehicles of different compounds, such as extracts or oils of various spices that have antioxidant and antimicrobial activity, as well as being friendly to the environment. The objective of this research was to develop a new edible coating based on chitosan enriched with peppermint extract and to evaluate its effectiveness to inhibit microbial development in vitro and improve both the quality and shelf life of common carp (*Cyprinus carpio*) during refrigerated storage (4 ± 1 °C). Three treatments were used: edible coating (C + EC), edible coating +, 5% chitosan (C + ECCh) and edible coating + 1.5% chitosan + 10% peppermint (C + ECChP). Prior the coating carp fillets; the antibacterial activity and antioxidant capacity were evaluated in the peppermint extract and coating solutions. After coating and during storage, the following were determined on the fillet samples: microbiological properties, observed for ECP, an inhibition halo of 14.3 mm for *Staphylococcus aureus*, not being the case for Gram-negative species, for ECCh, inhibition halos of 17.6 mm, 17.1 mm and 16.5 mm for *S. aureus, Salmonella typhimurium* and *Escherichia coli,* respectively; for the ECChP, inhibition halos for *S. aureus, S. typhimurium* and *E. coli* of 20 mm, 17 mm and 16.8 mm, respectively. For the physicochemical characteristics: an increase in solubility was observed for all treatments during storage, reaching 46.7 mg SN protein/mg total protein for the control, and values below 29.1 mg SN protein/mg total protein (*p* < 0.05), for fillets with EC (C + EC > C + ECCh > C + ECChP, respectively at the end of storage. For the pH, maximum values were obtained for the control of 6.4, while for the fillets with EC a maximum of 5.8. For TVB-N, the fillets with different CE treatments obtained values (*p* < 0.05) of 33.3; 27.2; 25.3 and 23.3 mg N/100 g (control > C + E C > C + ECCh > C + ECChP respectively). Total phenolic compounds in the aqueous peppermint extract were 505.55 mg GAE/100 g dried leaves, with 98.78% antioxidant capacity in the aqueous extract and 81.88% in the EC. Biomolecule oxidation (hydroperoxide content) had a significant increase (*p* < 0.05) in all treatments during storage, 1.7 mM CHP/mg protein in the control, to 1.4 in C + EC, 1.27 in C + ECCh and 1.16 in C + ECChP; TBARS assay values increased in the different treatments during refrigerated storage, with final values of 33.44, 31.88, 29.40 and 29.21 mM MDA/mg protein in the control; C + EC; C + ECCh and C + ECChP respectively. In SDS -PAGE a protective effect was observed in the myofibrillar proteins of fillets with ECChP). The results indicate that the C + ECCh and C + ECChP treatments extend the shelf life of 3–5 days with respect to microbiological properties and 4–5 days with respect to physicochemical characteristics. A reduction in lipid and protein oxidation products was also observed during refrigerated storage. With these findings, this is considered a promising method to increase the shelf life of fish fillets combined with refrigeration and we are able to recommend this technology for the fish processing industry.

## 1. Introduction

Biopolymers have become an alternative for synthetic packaging that is non-biodegradable and has a negative impact on the environment. Biopolymer-based edible coatings (ECs) can increase the shelf life and quality of food, acting as selective barriers against humidity and oxygen, lipid oxidation and loss of volatile aromatic compounds [1,2]. Commonly used polymeric materials include polysaccharides and proteins. Chitosan is a cationic polysaccharide obtained from crab exoskeleton, has several polar groups such as –OH and –NH2 which can act as electron donors, composed of (1, 4)-2-amino-deoxy-beta-D-glucan, the deacetylated form of chitin. Because of its non-toxic character, antifungal and antimicrobial activity, biodegradability, and biocompatibility as well as film-forming and antioxidant properties, chitosan has been widely applied in the preservation of meat products such as beef, chicken, mutton and fish [3,4,5,6,7,8,9,10].

Fresh fish is highly perishable as a result of its biological composition. Decomposition of fish muscle is the result of biological reactions such as lipid and protein oxidation which are due to the enzymatic activity characteristic of the species or the metabolic activity of the microorganisms present. In either case, muscle decomposition leads to shelf-life reduction in fish and fish products [11,12,13,14]. Numerous studies indicate that the use of chitosan reduces such reactions [6,11,14,15,16,17]. Rajalakshmi et al. [18,19] demonstrated that the extracted chitosan exhibits potent antioxidant activity and free radical scavenging activity, including activity toward DPPH radicals, hydrogen peroxide and superoxide anion radicals, and López-Caballero et al. [4] concluded that the chitosan-gelatin solution employed allowed cold preparation of a coating that was suitable for preventing fish spoilage, Kanatt et al. [19] suggested using peppermint extracts as coadjuvants in this activity.

*Mentha piperita* L. is a medicinally valuable plant of the family Lamiaceae and is commonly known as peppermint. It is a hybrid of *M. spicata* L. (spearmint) and *M. aquatica* (water mint). At present, it is grown in temperate zones in almost all regions of the world [20]. It has antiviral and antimicrobial activity, is a strong antioxidant, has antitumoral, antispasmodic, anti-inflammatory and antiseptic action, and anti-allergenic potential. The volatile oil of peppermint is composed mainly of menthol (29–48%), menthone (20–31%), menthofuran (6.8%) and menthyl acetate (3–10%). Other active compounds include bitter substances such as caffeic acid, flavonoids (12%), polymerized polyphenols (19%), carotenes, tocopherols, betaine, choline, quinones and tannins, the latter being present in aqueous extracts with antioxidant activity [20,21,22], Liu et al. [23] observed that chitosan-based films to which are added peppermint extract oil can significantly enhance the barrier, physicochemical, and antioxidant properties of the composite films, additionally Kavas et al. [24], demonstrated that mint extract introduced excellent antioxidant activities and showed antibacterial activity against Gram-positive food pathogens in chitosan added film.

The development of ECs enriched with natural preservatives that have both antibacterial and antioxidant activity and extend the shelf life of fresh fish is of great interest. The present study aimed to determine the effects of chitosan-based ECs enriched with aqueous peppermint extract—which may extend the quality of fillets of common carp (*Cyprinus carpio*), a valuable fish farm species worldwide—through the evaluation of antioxidant and antibacterial activity.

## 2. Materials and Methods

### 2.1. Materials

Chitosan of medium molecular weight, deacetylation value 75–85%, and viscosity 200–800 cP, was purchased from Aldrich Chemical. Gelatin, is derived from porcine skin, is soluble in hot rather than in cold water. It is practically insoluble in most organic solvents. The Bloom number, determined by the Bloom gelometer, is an indication of the strength of a gel formed from a solution of known concentration. The Bloom number is proportional to the average molecular mass. This product has a gel strength of 250, and was purchased in Sigma-Aldrich (Toluca, Mexico). Inulin from chicory plant (Frutafit IQ, VA Mexico SA CV).

Bovine serum albumin, 2,2-diphenyl-1-picrylhydrazyl (DPPH), acrylamide, N,N’-methylenebisacrylamide, trichloroacetic acid (TCA), FeSO4, sulfuric acid, cumene hydroperoxide (CHP), butylhydroxytoluene, methanol, xylenol orange, di-nitrophenylhydrazine (DNPH), guanidine, ethanol, ethyl acetate, hydrochloric acid, Coomassie Brilliant Blue R-250, thioglycolic acid, 4-chloro-7-nitrobenzofurazan (NBD-Cl), and o-phthalaldehyde (OPA) were obtained from Sigma-Aldrich (St. Louis, MO, USA).

Thiobarbituric acid (TBA) was purchased from Fluka (Sigma-Aldrich, Toluca, Mexico); sodium chloride, EDTA disodium salt, N,N,N′,N′- tetramethylethylenediamine (TEMED), Tris Base, urea, β-mercaptoethanol, glycine, glacial acetic acid, monobasic sodium phosphate, dibasic sodium phosphate and copper sulfate pentahydrate from J.T. Baker (Pennsylvania, PA, USA); sodium carbonate and lactic acid from Fermont (Monterrey, Mexico); sodium dodecyl sulfate (SDS) and bromophenol blue from Hycel (Mexico City, Mexico) and plate count agar from Bioxon (Becton Dickinson, Mexico City, Mexico). All reagents used were of analytical grade.

#### 2.1.1. Fish Sample Preparation

**Figure 1:**
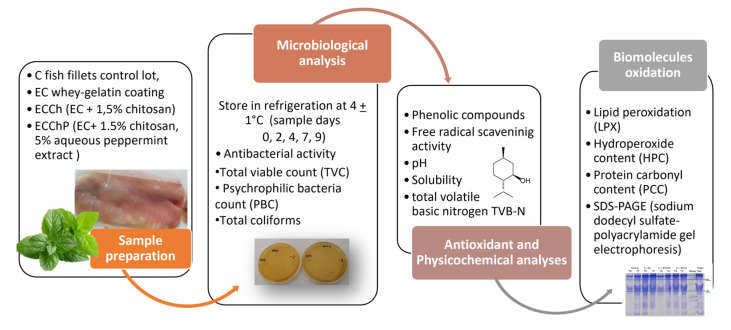
Representation of methodology.

On Figure 1, general methodology is shown. Fresh specimens of common carp (*Cyprinus carpio*) with an average weight of 550–650 g were purchased from Centro Acuícola Tiacaque in Toluca (State of Mexico), taken to the Food Laboratory in the Chemistry Department at Universidad Autónoma del Estado de México, and filleted by hand. Fish were harvested during July 2016. Fillets were obtained after removing the head and bones, and immediately immersed in the coating solutions.

#### 2.1.2. Extract with Antioxidant Activity

Aqueous extract of peppermint was prepared from leaves purchased at the Morelos Market (Toluca). Dried leaves were placed on trays and exposed to UV light for approximately 6 h to reduce the bacterial load. Next, 25 g of chopped, dried leaves were infused in 100 mL distilled water, and left at ambient temperature for 12 h, stirring occasionally by hand.

#### 2.1.3. Preparation for Edible Coating (EC) Solutions and Treated Fillets

EC solution base was prepared according to the formulation in García-Argueta et al. [25], consisting in 13% whey, 6% gelatin, 13% glycerol, 4% inulin and 1.5% lactic acid. The solution was stirred constantly at 40 °C until all components were fully dissolved, the final pH being 3.5.

Fillets were assigned at random to four lots: an uncoated control lot (C), a second lot coated with the EC base (C + EC), a third lot coated with the EC base + 1.5% chitosan (C + ECCh), and a final lot coated with the EC base + 1.5% chitosan and 5% aqueous peppermint extract (C + ECChP).

Approximately 15 fillets (12–15 cm) were used per lot. The EC was applied by immersion in the coating solution for 15 s, after which fillets were placed on a mesh to drain and air-dry for 1 min, enabling film formation, then stored at 4 ± 1 °C in a commercial refrigerator (Mabe, Mexico City, Mexico) for nine days to carry out subsequent analyses. Microbiological and physicochemical analyses were conducted on days 0, 1, 3, 7 and 9 to determine fish quality during storage.

### 2.2. Microbiological Analyses

#### 2.2.1. Determination of Antibacterial Activity

##### Bacterial Strains

One Gram-positive strain (*Staphylococcus aureus* ATCC 25923) and two Gram-negative strains (*Salmonella typhimurium* ATCC 14,028 and *Escherichia coli* ATCC 25922) were used to test the antimicrobial activity, as these are pathogenic bacteria identified in humans and transmitted by foods [14,26,27]. Bacterial cultures were maintained on agar at 4 °C during the study and were used as stock cultures.

#### 2.2.2. Antibacterial Activity

Bacterial suspensions were prepared as described by Singh et al. [20]. Turbidity was measured in a Genesys 10S Vis spectrophotometer (Thermo Scientific, Madison, WI, USA) at 610 nm and compared to a BaSO_4_ 0.5 McFarland standard suspension equivalent to 1.5 × 108 cells/mL. The suspensions were spread on Muller–Hinton agar, and cellulose circles (5 mm diameter) imbued with peppermint extract or coating solutions were placed on top. The plates were incubated at 35 °C for 18–22 h. Antibacterial activity was determined by the width of the zone of inhibition (clear of growth), and results were expressed as inhibition zone diameter. All tests were performed in triplicate.

#### 2.2.3. Bacteriological Analyses

The plate count method was used. Fish fillet (10 g) was homogenized with 90 mL of 0.1% peptone. Decimal dilution series were prepared from the latter dilution and placed in Petri dishes containing plate count agar. These inoculated plates were incubated at 35 °C for 48 h to determine total viable count (TVC), and at 0 °C for 7 days to determine psychrophilic bacteria count. To determine total coliforms, inoculated plates were added violet red bile agar (VRBA) and incubated at 36 °C for 24 h. Counts were expressed as log10 CFU/g, and were carried out in duplicate [28,29].

### 2.3. Antioxidant Capacity

#### 2.3.1. Determination of Total Phenolic Compounds

The total content of phenolic compounds was determined by the method described in Gao et al. [30]. To 100 μL of peppermint extract or coating solution in a test tube was added 700 μL of a 0.2 N solution of Folin–Ciocalteu reagent. Test tube contents were mixed and left to rest for 3 min at 25 °C, then supplemented with 900 μL sodium carbonate, and the mixture left for 90 min in the dark, following which absorbance was determined at 765 nm with the spectrophotometer. Total phenolic compounds were expressed as mg gallic acid equivalent (GAE)/g, based on a type curve, from 32 to 244 μg of gallic acid.

#### 2.3.2. Free-Radical Scavenging Activity

Antiradical activity was determined by the method proposed by Ranilla et al. [31], with modifications: 100 μL of peppermint extract or coating solution (at a concentration of 0.7 mg/mL total phenolic compounds) was transferred to conical-bottom polypropylene tubes, supplemented with 2.8 mL DPPH (98.9 μM in methanol) and Vortex-shaken for 15 s. The tubes were left to rest in the dark for 30 min, after which absorbance was read at 515 nm. Methanol was used as the reaction blank and Trolox solution (0.02 mM) as the antioxidant control sample. Antioxidant activity was expressed as the percentage of inhibition:

% inhibition = [(A0_515nm_-At_515nm_)/A0_515nm_] × 100 (1)

where: A0_515nm_ = absorbance in absence of the extract or coating solution, and At_515nm_ = absorbance in presence of the extract or coating solution.

### 2.4. Physicochemical Analyses

#### 2.4.1. Myofibrillar Protein Extraction

Myofibrillar protein (MP) was obtained according to the methodology described by Ngapo et al. [32], with slight modifications. Fish muscle (100 g) was homogenized in a blender for 10 min with ice and cold water in a 1:1:1 (*w*/*w*/*v*) ratio, then placed for 10 min in an ice bath with a magnetic stirrer. The myofibrillar suspension was strained twice through two cheesecloth layers to remove connective tissue. The homogenate was centrifuged at 3000× *g* and 4 °C for 25 min and the supernatant was discarded. Protein content in the myofibrillar precipitate was determined by the biuret method [33], and 25 mg/mL of MP was stored in a lidded jar for gel formation. The gelation process was carried out in two steps: first incubating at 4 °C for 60 min, then heating in a water bath with constant stirring and gradual temperature increases for 20 min until 90 °C was reached. The lidded jars were then removed and stored at 4 °C for later analysis.

#### 2.4.2. Solubility

As per Pilosof [34], 2 g of MP were centrifuged at 2500× *g* and 4 °C for 30 min. Protein content was determined in the supernatant, and total protein content in the MP sample prior to centrifuging. Solubility was determined by the ratio: protein content in the supernatant to total protein content in the MP sample, ×100, and was expressed as mg supernatant (SN) protein/mg total protein.

#### 2.4.3. pH

As described by Owen et al. [35], to 10 g of fish muscle was added 90 mL distilled water prior to homogenizing for 1 min in the blender. Connective tissue was removed by straining through cheesecloth. A digital potentiometer (Conductronic PH120, Mexico City, Mexico) was used to determine pH.

#### 2.4.4. Determination of Total Volatile Basic Nitrogen (TVB-N) Content

To quantify TVB-N content, the method of Conway and Byrne [36] was used, with slight modifications. To 5 g of homogenized fish fillet, was added 4% TCA in a 1:2 (*w*/*v*) ratio, and the resulting mixture was filtered through Whatman No. 1 paper (Schleicher & Schuell, Maidstone, UK). Filtrate (1 mL) was transferred into the outer ring of the Conway unit, and a 1% boric acid solution containing Shiro-Tashiro indicator was pipetted into the inner ring. To start the reaction, 2 mL K_2_CO_3_ were mixed with the filtrate. The unit was incubated at 25 °C for 24 h. The inner ring solution was titrated with 0.1 M HCl until a color change to pink ensued. Results were expressed as mg N/100 g fish muscle.

### 2.5. Biomolecules Oxidation

#### 2.5.1. Determination of Lipid Peroxidation (LPX)

To determine LPX the technique described by Büege and Aust [37] for thiobarbituric acid reactive substances (TBARS) was used. To a 100-µL aliquot of supernatant obtained from previously deproteinized fish fillet, Tris-HCl buffer solution pH 7.4 was added to attain a final 1 mL volume. The samples were incubated at 37 °C for 30 min, then supplemented with 2 mL TBA-TCA reagent (0.375% TBA in 15% TCA) and homogenized in a Vortex shaker. The homogenate was heated to boiling point in a hot water bath for 45 min, left to cool, centrifuged at 3000× *g* for 10 min and the resulting precipitate discarded. Absorbance was determined at 535 nm against a reaction blank. Malondialdehyde (MDA) content was calculated using the molar extinction coefficient (MEC) of MDA (1.56 × 105 M/cm). Results were expressed as mM MDA/mg protein.

#### 2.5.2. Determination of Hydroperoxide Content (HPC)

HPC was determined by the ferrous oxidation-xylenol orange (FOX) method of Jiang et al. [38]. Fish fillet samples were deproteinized with 10% TCA. To 100 µL of the resulting supernatant was added 900 µL of a reaction mixture [25 mM H_2_SO_4_, 0.25 mM FeSO4, 0.1 mM xylenol orange and 4 mM of 90% butylhydroxytoluene (*v*/*v*)] and the resulting mixture was incubated for 60 min at ambient temperature. Absorbance was read at 560 nm against a reaction blank. Results were interpolated on a previously constructed type curve and were expressed as nM cumene hydroperoxide (CHP)/mg protein.

#### 2.5.3. Determination of Protein Carbonyl Content (PCC)

The method of Levine et al. [39] was used, as modified by Parvez and Raisuddin [40] and Burcham [41]. To a 100-µL aliquot of the supernatant obtained from a fish fillet sample deproteinized with 10% TCA, was added 150 µL of 10 mM DNPH dissolved in 2 M HCl, and the resulting mixture was left at ambient temperature in the dark for 1 h. To stop the reaction, 500 µL of 20% TCA was added and the mixture was left to rest for 15 min at 4 °C. A precipitate was obtained by centrifuging at 11,000× *g* for 5 min; this was washed thrice with 1:1 ethyl acetate:ethanol solution. To dissolve the bud, 1 mL of 6 M guanidine pH 2.3 was added and the resulting mixture left for 30 min at 37 °C. Absorbance was read at 366 nm. The MEC of 21,000 M/cm was used. Results were expressed as nM reactive carbonyls formed (C=O)/mg protein.

#### 2.5.4. Total Sulfhydryl (SH) Content

Total SH content was quantified according to Ellman [42]. To 1 mL of MP solution at a concentration of 5 mg/mL, was added 9 mL Tris-glycine buffer (10.4 g Tris-HCl, 6.9 g glycine, 480 g urea and 1.2 g EDTA/L at pH 8.0) and the resulting mixture was maintained at ambient temperature for 30 min. A series of three tubes with 3 mL aliquots of this reaction mixture were supplemented with 0.05 mL Ellman’s reagent (4 mg DTNB/mL), incubating for 30 min in the dark. Absorbance was determined at 412 nm using a spectrophotometer. Total SH content was expressed as μM total SH/mg protein.

#### 2.5.5. SDS-PAGE (Sodium Dodecyl Sulfate-Polyacrylamide Gel Electrophoresis)

Gel electrophoresis was performed according to Laemmli’s [43] method in a system with a Mini-PROTEAN II electrophoresis cell (Bio-Rad, Hercules, CA, USA), using 10% acrylamide. To MP extracts were added 10% urea and sample buffer [0.1 M Tris-HCl (pH 6.8), 0.4% SDS, 10% glycerol and 0.004% bromophenol blue]. The running gel (140 × 140 mm) was fixed at 10%T in 1.2 M Tris-HCl (pH 8.8) and 0.3% SDS; and the stacking gel at 4%T in 0.25 M Tris-HCl (pH 6.8) and 0.2% SDS. The electrode buffer contained 0.025 M Tris-HCl, 0.192 M glycine and 0.15% SDS at pH 8.16. Electrophoresis was performed at 200 V. After the run, gels were stained with a solution of 40% methanol, 15% acetic acid and 0.1% Coomassie^®^ Brilliant Blue R-250.

### 2.6. Statistical Analysis

All statistical tests were performed in triplicate and a fully randomized design was used. All data were statistically analyzed with SPSS/PC v17 software. One-way analysis of variance (ANOVA), and independent samples and paired Student’s *t*-tests were used for comparison of means.

## 3. Results and Discussion

### 3.1. Microbiological Analyses

#### 3.1.1. Determination of the Antibacterial Activity of Peppermint Extract and Coating Solutions

**Figure 2:**
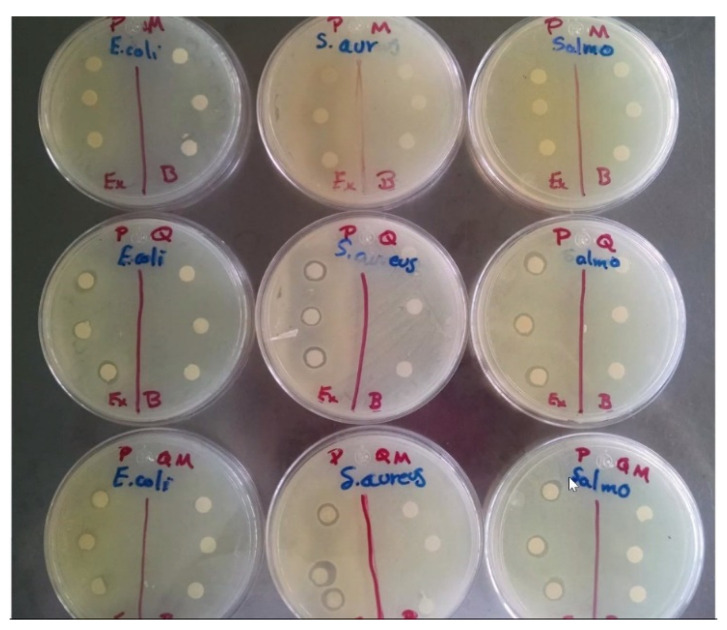
Halos of Inhibitory zone of P = EC; Q = ECCh; M = ECP; QM = ECChP.

Figure 2 shows the results of bacterial activity. In the case of ECP, sensitivity to *S. aureus* was observed, with a halo of inhibition of 14.3 mm that was not present in Gram-negative species. ECCh evidenced sensitivity to both groups of bacteria, with halos of 17.6 mm for *S. aureus*, followed by *S. typhimurium* (17.1 mm) and *E. coli* (16.5 mm). In ECChP, a synergistic effect was observed on *S. aureus* since a large zone of growth inhibition (20 mm) was found, unlike *S. typhimurium* and *E. coli* which had inhibition zones of 17 to 16.8 mm. The low levels of Gram-negative species inhibition by ECP may be due to the presence of lipopolysaccharides in the external membrane of these bacteria, which permit increased resistance to the antibacterial substances present in the aqueous peppermint extract [20]. As regards Gram-positive species, Al-Hadi [44] mentions that the effect of the latter extract on *S. aureus* is due mainly to the compounds 1,8-cineole, eugenol, sabinene and 4-terpineol, which show antibacterial activity as a result of their capacity to inactivate microbial and cell envelope proteins. In the case of ECCh, Dutta et al. [5] suggest that the antimicrobial character of chitosan is due to interaction of positive charges in the amine group with negative charges in the cell membrane of bacterial species. Zheng and Zhu [45] state that the mechanism of action of chitosan differs in Gram-positive and Gram-negative species. In *S. aureus*, antimicrobial activity may have increased due to the degree of deacetylation of chitosan, which can form on the cell surface a polymeric membrane inhibiting nutrient entry into the cell. As the degree of deacetylation decreases, antimicrobial activity against Gram-negative species increases, resulting in low molecular weight chitosan entering the cell through diffusion. In our study, the use of chitosan with a higher degree of deacetylation showed low antimicrobial activity against Gram-negative species (*S. typhimurium and E. coli*), this finding matches with previous works in which Gram-negative bacteria seemed to present higher resistance against chitosan [26], due to the cell wall lipopolysaccharides [46]. The antimicrobial effect of chitosan increases in the presence of extracts, as reported by Dutta et al. [5], Singh et al. [20] demonstrated that mint extracts do act as antimicrobial on *S. aureus*, in the present study, ECChP (the chitosan–peppermint mixture) had a synergistic effect on *S. aureus*, as showed in Figure 2, where halos were observed.

#### 3.1.2. Bacteriological Analyses

**Figure 3:**
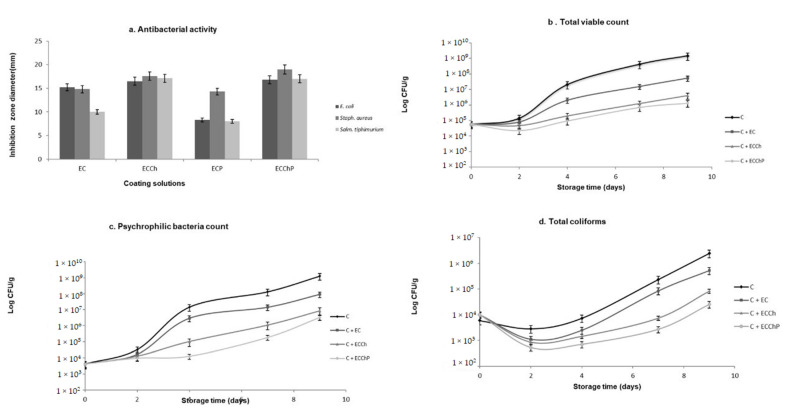
Changes in the microbiological characteristics of: (**a**) peppermint extract and coating solutions; and (**b**–**d**) common carp (Cyprinus carpio) fillets stored at 4 °C for nine days. Values are the mean of three replicates. C = uncoated fillet (control); C + EC = carp fillet with edible coating; C + ECCh = carp fillet with chitosan-based coating; C + ECChP = carp fillet with chitosan-based coating enriched with peppermint extract; CFU = colony-forming units.

Results of bacteriological analyses are shown in Figure 3b. TVC variations were observed during refrigerated storage. Basal values (Log10 CFU/g) for carp fillet treatments were 4.7. In control group fish muscle (C), TVC had increased to 7.3 on day 4, reaching the maximum recommended limit of 7 Log10 CFU/g in raw fish [29], while in treatment C + EC, this limit was reached on day 7, and in C + ECCh and C + ECChP on day 9 indicating a five-day extension of shelf life. Psychrophilic bacteria count increased from day 2 on (Figure 3c), with the highest increase (9.1) occurring in the control group, while fish fillets with chitosan-based ECs reached 6.9 (C + ECCh) and 6.5 (C + ECChP). As regards total coliforms (Figure 3d), the control group reached 6.3 Log10 CFU/g at the end of the storage period, while fish fillets with EC remained below this value, with treatment C + ECChP recording the lowest value of all (4.4 Log CFU/g).

The extended shelf life using edible coatings with essential oils is consistent with several authors [6,14,16]. Ojagh et al. [6], reported the effect of chitosan coating enriched with cinnamon indicating an extended microbiological shelf life over day 8 of storage on rainbow trout; Li et al. [16], observed that the recommended limit was reached at 16 days of storage using chitosan-tea polyphenols.

Psychrophiles are among the major microorganisms eliciting damage to fish in refrigerated storage [29]. Ramezani et al. [47], observed that psychrophiles of silver carp fillets treated with chitosan reached the maximal permissible limit of 7.0 log10 CFU/g three days later than the control consistent with behavior in this study.

Chitosan is widely known for its antimicrobial properties, which may be due to interaction between positive charges in the molecule and negative charges in the microbial membrane, inducing leakage of cell proteins and other intracellular constituents [48], or it could be an effect inside the cell membrane, where there is inhibition of enzymes activity, as well as RNA or DNA synthesis [7,16,17,49]. Also, chitosan-based ECs act as an oxygen barrier that inhibits growth of aerobic bacteria, while peppermint extracts act as coadjuvants in inhibition of bacterial growth since the presence of the latter extract induced reductions in bacterial counts during storage (Figure 3b). According to Al-Hadi [44], this may be due to the peppermint components present in the aqueous extract (menthol, menthone and certain tannins), which may act against microorganisms by acting over cell membranes phospolipds, increasing cell permeability and, therefore, origin intracellular constituents leakage as well as suppress microbial enzyme systems [7,17].

Additionally, the reductions in TVC, psychrophilic bacteria count and total coliforms evidenced in treatment C + EC may be due to the whey component in EC. Motalebi and Seyfzadeh [50], observed such antimicrobial activity in Gram-positive and Gram-negative species. Ju et al. [51], found that various ECs incorporated with essential oils (EOs) have stronger antimicrobial activity, as well as a longer duration than free EOs, and this could be because formulation and the surface charge of the EC could affect the mechanism of EOs in the cell membrane; also, the use of large molecules may help to improve this activity. In many cases, the EC with EOs can interact with multiple molecular sites on the microbial cell membrane.

### 3.2. Physicochemical Analyses

#### Solubility, pH and TVB-N Content

**Figure 4:**
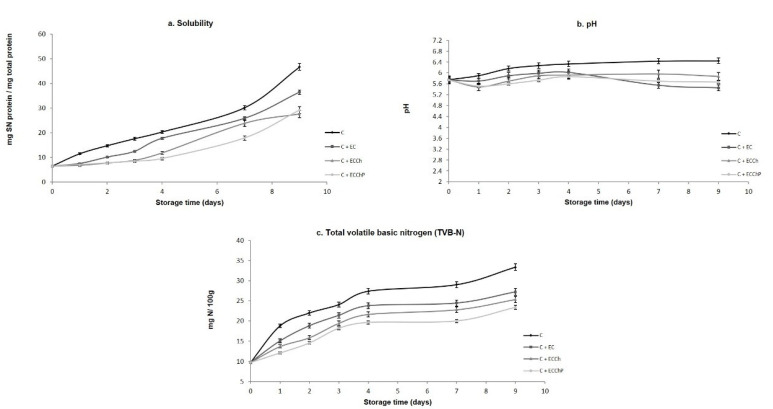
Changes in the physicochemical properties of common carp (*Cyprinus carpio*) fillets stored at 4 °C for nine days. Values are the mean of three replicates. C = uncoated fillet (control); C + EC = carp fillet with edible coating; C + ECCh = carp fillet with chitosan-based coating; C + ECChP = carp fillet with chitosan-based coating enriched with peppermint extract; SN = supernatant.

Near the isoelectric point (pI) of a protein, solubility generally increases with hydrolysis, since it is mainly the result of molecular weight reduction and increase in the number of polar groups [52]. In the present study, all treatments showed increased solubility during storage (Figure 4a), such increases being higher in uncoated fish fillets (C) which reached a value of 46.7 mg SN protein/mg total protein. Significantly lower values (*p* < 0.05) were found in fillets with EC (C + EC > C + ECCh > C + ECChP respectively), the C + ECChP treatment attaining 29.1 mg SN protein/mg total protein at the end of the storage period. This increased solubility may be due to weakening of fibrous linkages in muscular structure [53]. Results of the present study show that independent of the type of coating, ECs have a protective activity on muscle proteins, with C + ECChP exerting the best protective effect during refrigerated storage, preventing loss of structural proteins. This may be due to decreased microbial metabolism and/or enzymatic activity.

Changes in pH values during the 9 days of cold storage are shown in Figure 4b, and basal pH in carp fillets was 5.74. Basal pH values differ depending on species, diet, season and stress levels at capture. In our study, pH values increased during storage (except on day 1) in fillets with EC remaining throughout at 5.4–6.0 but ranged from 5.8 to 6.4 in uncoated fillets. A similar trend was observed by Sun et al. [54] and Yu et al. [55] after 3 days of grass carp (*Ctenopharyngodon idellus*) fillets cold storage. This may be due to the pH value of the film itself, since the gradual pH increase can be attributed to volatile base increases [15,16,56], as well as to the formation of alkaline substances, such as ammonia, biogenic amines and, trimethylamines, caused by microorganisms and endogenous enzymes, contributed to the increase in pH values [57], reflected in Figure 4c.

The maximum permissible value for volatile base parameters is 25 mg N/100 g fish muscle, as proposed by Giménez et al. [58]. In the present study, TVB-N content increased from a basal value of 9.7 to 33.3 in the control group (C); similar results were found by Wang et al. [57] in fresh-salmon fillets found 45 mg N/100 g after 9 days of refrigeration storage in control, and the samples with collagen-lysozyme coating reaching values of 20 mg N/100 g to 9 days of storage. Alsaggaf et al. [17] in Nile tilapia fillet found 60 mg N/100 g after 30 days of storage, using chitosan at 2% incorporated with pomegranate peel as an edible coating observed increments on the TVB-N values of 12–22.3 mg N/100 g; in our study, values were 27.2 in C + EC, 25.3 in C + ECCh and 23.3 in C + ECChP on day 9 of storage, so that fillets with chitosan-based ECs avoided deterioration for up to five additional days. Production of ammonia, dimethylamine, trimethylamine, and formaldehyde is caused by spoilage bacteria, autolytic enzymes, and deamination of amino acids as well as nucleotide catabolites, so that high TVC values favor formation of these compounds. In the present study, fillets with ECs had lower TVC values and, therefore, volatile base production was lower in these treatments [6,59,60].

### 3.3. Antioxidant Capacity

Total phenolic compounds in the aqueous peppermint extract were 505.55 mg GAE/100 g dried leaves, with 98.78% antioxidant capacity observed in the aqueous extract and 81.88% in the EC. Comparison of antioxidant activity, though reported in diverse studies [20,61,62,63], is difficult, since data are significantly affected may be due to different agro-climatic (climatic, seasonal and geographical) variations, extraction procedures and physiological conditions of the plants [63]. Elansary et al. [61] reported that in methanolic extracts of *Mentha piperita* and *Menta longifolia* from Saudi Arabia were detected six phenolic acids, and the major polyphenol in *M. longifolia* was rosmarinic acid (781.6 mgGAE/100 g); Singh et al. [20] studied extracts of *Mentha piperita* from Libya, which showed aqueous extract scavenging activity of 70.3%; Farnad et al. [62] observed that the extracts of various alcoholic solvents have different levels of antioxidant activity in Peppermint (*M. piperita*) growing in Iran, where the methanol extract had a maximum content of 3.57 mg GAE/100 g; Tahira et al. [63] studied several mint species methanolic extracts from different origin (Islamabad, China, Canada and Azad Jammu and Kashmir) observed that they exhibited a wide range of phenolic acid profiles and concentrations, with the highest concentration the rosmarinic acid (*Mentha arvensis* with 362.2 mg/100 g; *Mentha spicata* had 298.7 mg/100 g and *Mentha piperita* 287.4 mg/100 g, respectively); however, our results are consistent with those of Kanatt et al. [19], who reported 85% inhibition, demonstrated the efficacy of chitosan and mint mixture as a potent antibacterial and antioxidant agent that can be used for the preservation and shelf life extension of meat and meat products; Singh et al. [20] who reported 70.3% inhibition in aqueous extract of *Mentha piperita*. Brown et al. [64] mentioned, in a discussion on polyphenol composition and antioxidant potential of mint leaves, that the total phenolic content is usually a good indicator of the antioxidant activity, and found a clear relationship between the total phenolic content and the antioxidant activity; samples with the higher phenolic content were more effective antioxidants, especially in the tests that measured free radical scavenging. Due to our results that showed total phenolic compounds and antioxidant capacity, we assume EC added with mint and chitosan could allow its use as a coadjuvant in prevention of oxidation.

### 3.4. Oxidation of Lipids and Proteins

**Figure 5:**
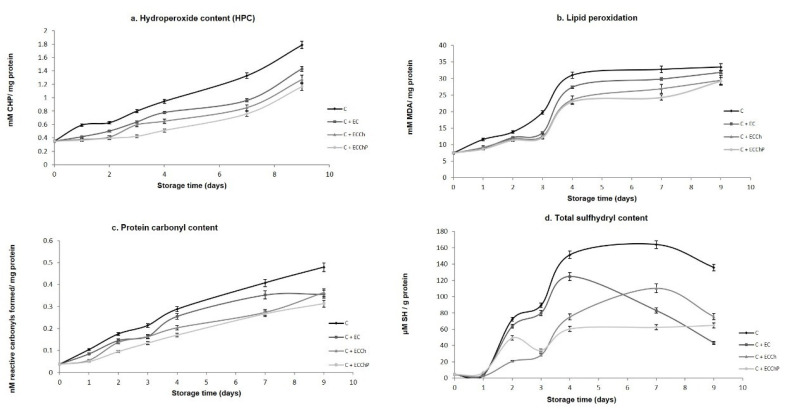
Changes in formation of oxidation products in common carp (Cyprinus carpio) fillets stored at 4 °C for nine days. Values are the mean of three replicates. C = uncoated fillet (control); C + EC = carp fillet with edible coating; C + ECCh = carp fillet with chitosan-based coating; C + ECChP = carp fillet with chitosan-based coating enriched with peppermint extract; MDA = malondialdehyde; CHP = cumene hydroperoxide; -SH = sulfhydryl.

Quantification of hydroperoxides is used to determine the formation of primary products of lipid oxidation during the storage period [15]. Figure 5a shows the effect of EC on HPC. Significant increases (*p* < 0.05) occurred in all treatments during storage, from 0.35 to 1.7 mM CHP/mg protein in the control group (C), to 1.4 in C + EC, 1.27 in C + ECCh and 1.16 in C + ECChP. Results showed that the chitosan-based EC and the chitosan-peppermint mixture were significantly effective (*p* < 0.05) in delaying hydroperoxide production during refrigerated storage, reducing lipid oxidation. Similar results were obtained with chitosan in other species (trout, herring and Atlantic cod) [6,15]. Ojagh et al. [6], observed the Ch + Cinnamon-coated samples showed a lower TBA and effective in retarding the production of PV in trout fillets stored by refrigeration (4 ± 1 °C) on rainbow trout fillets; Jeon et al. [65], who reported that chitosan coating was effective in retarding the production of primary lipid oxidation products in herring and Atlantic cod fillets stored at 4 ± 1 °C. Chitosan coatings show good barrier properties against oxygen permeability, delaying the diffusion of ambient oxygen towards the surface of fish flesh [11].

TBARS assay values are widely used to determine the degree of lipid peroxidation during the second phase of autooxidation in which peroxides and hydroperoxides are oxidized to aldehydes and ketones. In the present study, TBARS assay values increased in the different treatments during refrigerated storage (Figure 5b), with final values of 33.44, 31.88, 29.40 and 29.21 mM MDA/mg protein in the control group (C) and treatments C + EC, C + ECCh and C + ECChP, respectively; the EC enriched with the chitosan–peppermint mixture showing the best protective effect. This may be due to the antioxidant mechanism of chitosan which forms a stable fluorosphere with aldehydes and the positive charges of the primary amine groups that compose chitosan in addition to acting as a chelating agent of metal ions, thus preventing lipid peroxidation. Also, the tannin content in aqueous peppermint extract can break the free-radical chain by donating a hydrogen atom, or its action as a free radical scavenger [6,7,17], exerting a synergistic effect on antioxidant activity. Mentha extracts can act against oxidative damage affording protection by eliminating iron (II) ions that could otherwise generate hydroxyl radicals through Fenton-type reactions or if the metal is found in a free non-sequestered form, catalyze decomposition reactions of hydroperoxide [66].

Intermediates of lipid oxidation and external factors such as manipulation, noise and stress can form reactive oxygen species that lead to oxidation of proteins, to form more carbonyl and sulfhydryl groups, which can affect the functional characteristics of the protein such as solubility and hydrophobicity [60,67] evidenced in this study through changes in disulfide groups and an increase in protein carbonyls groups (Figure 5c,d). Total SH group content indicates protein conformational changes during cold storage; a reduction in these indicates disulfide bond formation [68]. A marked decrease in SH content was observed after day 4 of storage (Figure 5c), the ECCChP having a superior protective effect and showing greater stability during storage, followed by the chitosan-based EC.

### 3.5. SDS-PAGE

**Figure 6:**
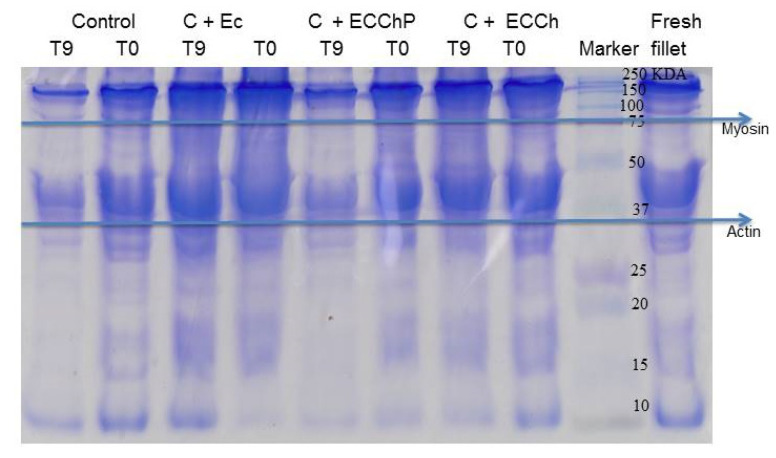
SDS-PAGE of MP during refrigerated storage. EC = edible coating base; ECCh = chitosan-based edible coating; ECChP = chitosan-based edible coating enriched with peppermint extract.

As the storage time is prolonged, the muscle tissues of the fish gradually soften and degrade, resulting in a decrease in the quality of the fish and the loss of its edible value. Proteins, the main components of muscle tissues, have the supporting structure and play an important role in various physiological and biochemical reactions in relation to the softening and spoilage of fish, so the degradation of proteins will have a direct impact on fish quality and become an important sign of the manifestation of fish damage [1,69]. In Figure 6, the SDS-PAGE electrophoretic profile of MP from *Cyprinus carpio* is shown under the different treatments EC = edible coating base; ECCh = chitosan-based edible coating; ECChP = chitosan-based edible coating enriched with peppermint extract. Characteristic bands of the heavy chain of myosin, actin and paramyosin of each sample are observed; all the characteristic proteins showed a tendency to degrade during the storage time. The upper bands (100–250 kDa) gradually widened, which could indicate that the heavy chain of myosin was degrading in proteins with lower molecular weights, the band of paramyosin disappeared in the control group, a behavior slower was observed in the treatments C + EC, C + ECCh and C + ECChP, so it could be a protective effect with the appearance of more defined bands. A similar trend was reported by Sun et al. [54], in grass carp fillets with fish gelatin coating enriched with curcumin/β-cyclodextrin, where they observed a decrease in myosin and paramyosin bands during storage time, presenting bands of low molecular weight. Therefore, myosin and paramyosin fragments could be an indicator of fish muscle deterioration, according to Sun et al. [54] observed during storage, the muscle tissues of the fish gradually soften and degrade, resulting in a decrease in the quality and edible value of meat, hence in our study, having a mixture of mint and chitosan, the synergistic effect could decrease the speed of oxidation reactions and growth of deteriorating microorganisms, since according to Sun et al. [70], observed that apple phenols incorporated into carp surimi could prevent the oxidation of sulfhydryl groups, and as a consequence a reduction of proteins degradation during refrigerated storage; likewise, Yu et al. [55] observed that chitosan coatings combined with monolauric glycerol, reduced the degree of chemical and microbial deterioration, therefore the softening of the texture was inhibited Kjærsgård et al., [71] mentioned that low abundant proteins could be relatively more carbonylated than very abundant proteins, indicating that some proteins are more susceptible to oxidation than others, due to their cellular location, amino acid sequence or biochemical function.

Therefore, protein degradation may be related to lipid oxidation products, since oxidative reactions can be easily transferred from lipids to proteins, due to the interactions that exist between these molecules, as well as the radicals, hydroperoxides and secondary compounds that result from the oxidation of lipids, which can also react with proteins, resulting in protein degradation, loss of texture and, as a consequence, loss of protein functionality [68,72,73,74,75]. On the other hand, Nie et al. [76] observed in SDS-PAGE that the band intensity of myofibrillar proteins in grass carp sausages decreased markedly or even disappeared throughout fermentation, due to proteolysis and acid induced denaturation, suggesting that bacteria can affect the breakdown of myofibrillar proteins. Therefore, the results of the chitosan coating added with mint could be a promising method to increase the shelf life of fish fillets combined with refrigeration.

## 4. Conclusions

In recent years, worldwide attention has been focused on the application of packaging that not only increases shelf life and maintains food quality but can also reduce environmental impact. In this study, the effects of the chitosan coating enriched with aqueous mint extracts on the quality of common carp (*Cyprinus carpio*) fillets were investigated, which maintained the physicochemical qualities (pH, solubility, total volatile basic nitrogen, sulfhydryl groups, oxidation lipid and protein) and microbiological quality of carp fillet samples, helping to extend their shelf life during storage at 4 °C; in general, ECChP coating treatment can increase the shelf life of carp by about 4 days compared with the control, making the fillets suitable for consumption. These results could be associated with the phenolic and structural components of both peppermint and chitosan, being that the concentrations used of each at 10% and 1.5%, respectively, were effective to obtain these benefits, hinting that it was a synergistic effect. Therefore more effective packaging with these findings could be a promising method to increase the shelf life of fish fillets combined with refrigeration and to consider this technology for the fish processing industry.

## Data Availability

All generated data in this study are included in the article.
